# On the privacy-conscientious use of mobile phone data

**DOI:** 10.1038/sdata.2018.286

**Published:** 2018-12-11

**Authors:** Yves-Alexandre de Montjoye, Sébastien Gambs, Vincent Blondel, Geoffrey Canright, Nicolas de Cordes, Sébastien Deletaille, Kenth Engø-Monsen, Manuel Garcia-Herranz, Jake Kendall, Cameron Kerry, Gautier Krings, Emmanuel Letouzé, Miguel Luengo-Oroz, Nuria Oliver, Luc Rocher, Alex Rutherford, Zbigniew Smoreda, Jessica Steele, Erik Wetter, Alex “Sandy” Pentland, Linus Bengtsson

**Affiliations:** 1 Department of Computing, Imperial College London, London SW7 2AZ, UK; 2 MIT Media Lab, 20 Ames St, Cambridge, MA 02139, USA; 3 Université du Québec à Montréal, Département d’informatique, Case postale 8888, succ. Centre-ville, Montréal (Québec), H3C 3P8, Canada; 4 Université catholique de Louvain, Place de l’Université 1, 1348 Louvain-la-Neuve, Belgium; 5 Telenor Research, Snarøyveien 30, 1360 Fornebu, Norway; 6 Orange, 44 avenue de la République, 92320 Châtillon, France; 7 Riaktr, 5 Place du Champs de Mars, 1050 Brussels, Belgium; 8 UNICEF, Office of Innovation, 3 UN Plaza, New York, NY 10017, USA; 9 University of Washington, Dept. of Computer Science, 708b 11th Avenue East, Seattle, WA 98102, USA; 10 Data-Pop Alliance, 99 Madison Avenue, 15th Floor, New York, NY 10016, USA; 11 UN Global Pulse, 370 Lexington Avenue, New York, NY 10017, USA; 12 Vodafone Research, Paddington Central, London, W2 6BY, UK; 13 University of Southampton, Geography and Environment, Building 44, University Road, Southampton, SO17 1BJ, UK; 14 Flowminder Foundation, Roslagsgatan 17, SE-11355, Stockholm, Sweden; 15 Stockholm School of Economics, Sveavägen 65, 113 83 Stockholm, Sweden; 16 Asian Institute of Management, 123 Paseo de Roxas, 1229 Metro Manila, Philippines

**Keywords:** Ethics, Society, Research data

## Abstract

The breadcrumbs we leave behind when using our mobile phones—who somebody calls, for how long, and from where—contain unprecedented insights about us and our societies. Researchers have compared the recent availability of large-scale behavioral datasets, such as the ones generated by mobile phones, to the invention of the microscope, giving rise to the new field of computational social science.

With mobile phone penetration rates reaching 90%^1^ and under-resourced national statistical agencies^2^, the data generated by our phones—traditional Call Detail Records (CDR) but also high-frequency x-Detail Record (xDR)—have the potential to become a primary data source to tackle crucial humanitarian questions in low- and middle-income countries. For instance, they have already been used to monitor population displacement after disasters^3^, to provide real-time traffic information, and to improve our understanding of the dynamics of infectious diseases^4^. These data are also used by governmental and industry practitioners in high-income countries.

While there is little doubt on the potential of mobile phone data for good, these data contain intimate details of our lives: rich information about our whereabouts, social life, preferences, and potentially even finances. A BCG study showed, e.g., that 60% of Americans consider location data and phone number history—both available in mobile phone data—as “private”.

Historically and legally, the balance between the societal value of statistical data (in aggregate) and the protection of privacy of individuals has been achieved through data anonymization. While hundreds of different anonymization algorithms exist, most of them are variations and improvements of the seminal *k*-anonymity algorithm introduced in 1998^5^. Recent studies have, however, shown that pseudonymization and standard de-identification are not sufficient to prevent users from being re-identified in mobile phone data. Four data points—approximate places and times where an individual was present—have been shown to be enough to uniquely re-identify them 95% of the time in a mobile phone dataset of 1.5 million people^6^. Furthermore, re-identification estimations using unicity—a metric to evaluate the risk of re-identification in large-scale datasets^6^—and attempts at *k*-anonymizing mobile phone data^7^ ruled out de-identification as sufficient to truly anonymize the data. This was echoed in the recent report of the [US] President’s Council of Advisors on Science and Technology on Big Data Privacy which consider de-identification to be useful as an “added safeguard, but [emphasized that] it is not robust against near-term future re-identification methods”.

The limits of the historical de-identification framework to adequately balance risks and benefits in the use of mobile phone data are a major hindrance to their use by researchers, development practitioners, humanitarian workers, and companies. This became particularly clear at the height of the Ebola crisis, when qualified researchers (including some of us) were prevented from accessing relevant mobile phone data on time despite efforts by mobile phone operators, the GSMA, and UN agencies^8^, with privacy being cited as one of the main concerns.

These privacy concerns are, in our opinion, due to the failures of the traditional de-identification model and the lack of a modern and agreed upon framework for the privacy-conscientious use of mobile phone data by third-parties especially in the context of the EU General Data Protection Regulation (GDPR). Such frameworks have been developed for the anonymous use of other sensitive data such as census, household survey, and tax data^9^. The positive societal impact of making these data accessible and the technical means available to protect people’s identity have been considered and a trade-off, albeit far from perfect^9^, has been agreed on and implemented. This has allowed the data to be used in aggregate for the benefit of society. Such thinking and an agreed upon set of models has been missing so far for mobile phone data^10^. This has left data protection authorities, mobile phone operators, and data users with little guidance on technically sound yet reasonable models for the privacy-conscientious use of mobile phone data. This has often resulted in suboptimal tradeoffs if any^8^.

In this paper, we propose four models for the privacy-conscientious use of mobile phone data (Fig. 1). All of these models 1) focus on a use of mobile phone data in which only statistical, aggregate information is ultimately needed by a third-party and, while this needs to be confirmed on a per-country basis, 2) are designed to fall under the legal umbrella of “anonymous use of the data”. Examples of cases in which only statistical aggregated information is ultimately needed by the third-party are discussed below. They would include, e.g., disaster management, mobility analysis, or the training of AI algorithms^11^ in which only aggregate information on people’s mobility is ultimately needed by agencies, and exclude cases in which individual-level identifiable information is needed such as targeted advertising or loans based on behavioral data.

First, it is important to insist that none of these models is a silver bullet. However, we believe that each one, depending on the stage of development of the project and the release cycle of the data, provides a reasonable balance between utility and privacy. They can all be used as a basis to use mobile phone data for positive social impact in a privacy-conscientious way, with costs deemed reasonable to telco’s data philanthropy efforts. Other models however also exist e.g. contractual arrangements that do not rely on anonymization including the pooling of data from several stakeholders through a trusted intermediary. We however do not discuss these models here as their privacy and security guarantees are non-technical and stem solely from contractual relationships between institutions. While our analysis and recommendations focus on mobile phone data, some of the challenges we highlight and the models we propose are likely to be applicable to other types of data. For instance, URL data were shown to have a high unicity^12^ making them likely to be re-identifiable, and the remote access model described below is used by the Secure Access Data Center (CASD) infrastructure in France to grant third-parties access to sensitive health data. Finally, our models focus on providing ways for mobile phone data to be anonymously used. Other risks related to ethics and membership inference attacks exist^13,14^. While such risks typically have to be legally addressed in data protection impact assessments (DPIA), some organizations go further, e.g., by setting up an external ethics committee to review data uses.

We will now review the four models, emphasizing their applicability to different data uses, pros and cons, and implementation challenges. We will also discuss potential threats and resulting information leaks for each model.

***Limited release*** is the closest model to traditional sharing of data. A mobile phone dataset is transformed in-house and a copy of the data is given to third-parties under a legal contract. The transformation aims at both adding technical difficulties to attempts at re-identifying individuals and at limiting the amount of information that could be uncovered if the data were to be re-identified. The transformed data are, however, still fairly close to the raw data. Transformations typically consist of 1) data sampling and longitudinal resampling with new identifiers - either using correspondence tables, properly salted hashes or the use of key-hash function - as well as 2) limited data coarsening along the temporal axis and Voronoi translation of antennas (spatial axis)^10^. We recommend limited spatial and temporal coarsening as it has been shown to only marginally help prevent re-identification while restricting the general useof the data^6^. Transformations affect, in general, the quality and quantity of the data available to researchers thereby limiting statistical power and potentially preventing important research questions from being explored. The main implementation challenge of the *limited release* model is probably the choice of the transformation. It requires an in-depth understanding of all of the current but also future uses of the data, as anonymization can usually only be performed once. Furthermore, as discussed before, these transformations alone are increasingly not sufficient to make the data subject “no longer identifiable” and, consequently, to release the data openly. Appropriate non-disclosure and data use agreements (DUA) are therefore required.

In the *limited release* model, the transformed data is released directly to the users. The data controller therefore loses technical control over the data. This significantly increases the risk of the data to be stolen, uploaded online, or to be part of a data breach. It puts a lot of weight on the data anonymization procedure. Because of this, we consider re-identification using auxiliary location information to be the main privacy threat in the *limited release* model: re-identification would allow an attacker to link the released data about one to all of the users back to their identities.

We therefore recommend the *limited release* model for data sharing with a small to medium-sized group of moderately trusted third-parties, for initial and exploratory data analysis. An example of *limited releases* are Orange’s D4D challenges, in which data were transformed (sampled, limited longitudinality, slightly coarsened, etc.) before being released to selected teams of researchers under strict DUA^15^.

***Pre-computed indicators and synthetic data.*** Despite the limits of data anonymization, there are cases in which one would like to (or has to by law) release data without restrictions on users or access. In the *pre-computed indicators* model, indicators derived from mobile phone data are released to third-parties. These indicators can be computed at individual level (e.g., number of calls, radius of gyration)^16^ or aggregated across individuals (e.g., number of users per tower over time, long or short-term mobility matrices, and matrices of inter-towers communications). Because indicators are 1) much more disconnected from both the raw and potential auxiliary data, and 2) potentially aggregated across individuals, they can often be properly anonymized. However, it should be noted that recent work in the privacy literature has started to question the level of protection that is really provided by aggregation methods^17^.

Similarly, synthetic data representations can be parameterized using mobile phone data and the parameters released openly along with the model. Synthetic data generated by the model and preserving pre-defined statistical properties of the original data can equivalently be released. However, little work, so far, exists in synthetic mobile phone data representations and the development of representative and useful synthetic data in other fields has proven challenging^18^.

On the privacy side, we see the main privacy threats for *pre-computed indicators and synthetic data* to be questions around the notion of “group privacy”^13,19^, which pertains to all release types. Definitions vary but, intuitively, the idea is that one’s individual privacy might be violated if information about a group he belongs to is revealed. Aggregated or anonymized data might indeed reveal sensitive information on groups and could lead to stigmatization or discrimination. In the case of mobile phone data, the privacy of a specific ethnic, or religious or minority group might, for example, be endangered if information about their behavior were to be revealed.

We therefore recommend the *pre-computed indicators* model for the open release of well-established and stable-across-time metrics of interest such as mobility and behavioral indicators for applicative purposes. Examples would include the release of flow maps parameterized using mobile phone data by Flowminder as part of the fight against Ebola^20^ or the release of tourism statistics by Statistics Netherlands^21^.

***Remote access*** is our first model using the privacy-through-security approach. Here, the data are not released but instead stay within the premises and under the control of the operator (or an authorized entity) and are analysed remotely. The data processing takes place within the operator’s premises and only aggregated data leave the secure area. In contrast to the data anonymization-based models we presented previously, the data controller does not have to relinquish all control over the data. The controller can supervise who accesses the data (having users registering, signing a DUA, setting restrictions on IP addresses), how the data are being used (e.g., through active monitoring of the secured environment or by controlling the output), and can ensure that no individual-level or raw data leave the server (through a manual approval process or by monitoring the amount of data leaving the server). While they do not remove all possible risks, these security-based mechanisms already strongly limit the risks of the data to be re-identified en masse and misused. This, in turn, allows the data controller to transform the data less aggressively, for instance only removing phone numbers and other direct personal identifiers, potentially along with limited temporal and spatial coarsening. This limited transformation as well as the ability to access data in near-real time strongly increase the utility and possible uses of the data.

We see the main privacy threat for the *remote access* model to be the risk of a targeted user to be re-identified. Because the data analysis happens within a secured and controlled environment, the mass re-identification of users and exfiltration of their data is very unlikely. A secondary threat would be for the server holding the data to be compromised. While not impossible, we do not consider this risk to be significantly higher than the risk of the server currently holding the data to be compromised. From a practical perspective, we see the funding and the development of such appropriately secured infrastructure—yet flexible enough to support a variety of research questions and tools—as the main practical challenge for the *remote access* model especially as it requires significant human investments from the telco.

We therefore recommend the *remote access* model to allow near real-time data to be used by highly-trusted third-parties under a DUA for confirmatory or applicative analysis including the training of AI algorithms. This is the model that was used by Flowminder when studying people’s mobility directly after the Nepal earthquake. A small number of registered researchers analysed pseudonymized mobile phone data remotely with security measures in place. CASD is an example of the type of infrastructure needed to support these kind of analyses. It allows researchers to access the data through a virtual desktop system with dedicated authentication hardware while any data taken out of the system are manually verified.

***Question-and-answer*** Last but not least, the *question-and-answer* (QA) model pushes the privacy-through-security approach one step further: the data stays within the premises of the operator but third-parties now only access the data through a question-and-answer system (e.g., SafeAnswers^22^ or SQL queries^23,24^). Questions are asked in the form of a piece of code whose answers are computed using the pseudonymized data. These are validated by the system before being sent back to the user through the API. Answers can be at the level of individuals or, more often, groups of individuals. A question could be, for example, “How many people have been travelling from city A to city B between this date and this date”. The results are then aggregated and validated, and the answer, e.g., “*3159*”, is shared with the third-party through the API. The same security mechanisms than for the *remote access* are put in place: registration of users, restrictions on IP addresses, etc. On top of this, because the framework and language used to ask the questions as well as the user-facing API are standardized, more advanced and automated security and auditing mechanisms can be put in place. For instance, the system can ensure that the code runs for each user independently within a sandbox and can, manually or automatically, validate it^25^. If answers are aggregated over groups of individuals, the system can also ensure that the aggregation mechanism protects individuals’ privacy ensuring, e.g., that *k* individuals have contributed to each answer, that a certain level of coarsening or noise addition is added, or guaranteeing differential privacy^26^. Finally, every question asked (both algorithm and parameters) can be fully logged.

In practice, the implementation details of these techniques will depend on the trust we place in users, how many users there are, and the estimated sensitivity of the data. We would consider reasonable a system with 1) some validation (manual or semi-automatic) of the code being used—potentially through a bank of open-source algorithms such as in the OPAL project—, 2) a strict control of the aggregation mechanisms used for each question, and 3) carefully added noise. If the data are distributed (across users or pieces of information), tools such as secure multiparty computation can be used to compute aggregated results^27^ or to run statistical analysis such as correlations^28^. We see the need for open-source software and practical privacy mechanisms to be the main challenges to the implementation of the *question-and-answer* model.

Since the use of the data is tightly controlled, we consider the server being compromised to be the main privacy threat. However, as for the *remote access* model, we do not consider this risk to be significantly higher than the risk of any places where the data would be digitally stored (server, laptops, etc.) to be compromised. While the likelihood of an attacker being able to infer information about a specific re-identified user through the QA API is not null (these attacks served as motivation for mechanisms such as differential privacy^26^), we consider this risk to be moderate when combined with defense-in-depth mechanisms. In both the *remote access* and *question-and-answer* model, the data controller does not lose technical control over the data and measures can always be taken as response to a potential privacy breach. We therefore recommend the *question-and-answer* model for more formalized uses of mobile phone data by a medium to high numbers of third-parties in near real or real time.

To conclude, mobile phone data has a great potential for good but its high dimensionality limits the applicability of traditional data anonymization methods. These limits have to be acknowledged and blanket anonymization or de-identification statements are not acceptable anymore. However, as recent crises have made abundantly clear, having qualified researchers being barred from accessing and using valuable mobile phone data is not acceptable either^8^. We have here proposed four models for the privacy-conscientious use of mobile phone data which we hope, moving forward, will help properly balance technically the need to use this data for good and the legitimate privacy concerns of individuals and societies.

## Additional information

**How to cite this article**: de Montjoye, Y.-A. *et al*. On the privacy-conscientious use of mobile phone data. *Sci. Data*. 5:180286 doi: 10.1038/sdata.2018.286 (2018).

**Publisher’s note**: Springer Nature remains neutral with regard to jurisdictional claims in published maps and institutional affiliations.

## Figures and Tables

**Figure 1:**
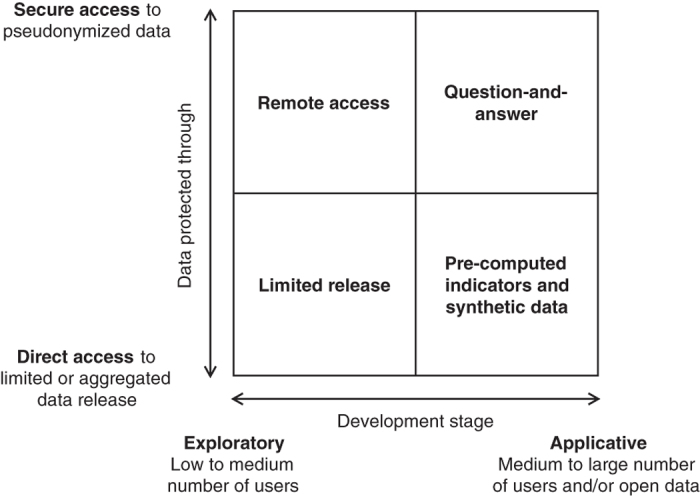
Matrix of the four models for the privacy-conscientious use of mobile phone data.
